# Audit of inpatient smoking cessation advice

**DOI:** 10.1192/bjo.2021.280

**Published:** 2021-06-18

**Authors:** Flensham Mohamed, Mohamed Bader

**Affiliations:** 1 Aneuran Bevan University Health Board; 2 Aneurin Bevan University Healthboard

## Abstract

**Aims:**

Audit carried out to assess whether or not patients had been asked about their smoking status during admission onto an acute adult mental health ward, as well as if they had received any smoking cessation advice or offered nicotine replacement therapy.

**Background:**

- Physical health outcomes in patients with serious mental illness (SMI) are consisitently worse than the general public This is due to multiple factors; adverse effects of medication (including metabolic syndromes with psychotropics) as well as poor lifestyle factors such as smoking status
- Patients with an SMI are 3–6 times more likely to die due to coronary artery disease. 70% of patients in inpatient psychiatric units are smokers, a strong independent risk factor for cardiovascular disease.
- Smoking cessation is a potent modifiable risk factor that can prevent mortality and reduce morbidity.

**Method:**

A cross-sectional review of all 34 inpatients across four general adult acute psychiatric wards.

Patient records were explored using the Aneuran Bevan Health Board admission proformas to identify evidence of smoking status and whether advice was offered.

**Result:**

Smoker but not given cessation advice n = 13 (38%)

Not asked about smoking n = 11 (32%)

Smoker and given cessation advice n = 4 (12%)

Non-smoker n = 6 (18%)

**Conclusion:**

Patients were asked about their smoking status the majority of the time (68%) but provision of advice or nicotine replacement therapy was only done in 14% of potential smokers (identified smokers and patients not asked about smoking status).

A consideration to be taken into account is that on admission, a patient's physical health status may be unknown, with the additional difficulty of a patient's acute distress complicating the physical examination, smoking status and modification of patient's smoking status may not be the highest priory in that context.

Data regarding asking about smoking were different amongst wards, potentially signifying differences between assessors willingness to ask about smoking status.

There is a lack of smoking cessation literature available on the wards and patients are often unaware of what options are available to quit smoking.

The audit simply determined whether or not assessors were documenting smoking status, it does not measure the quantity or quality of smoking cessation advice provided.

Further quality improvement projects should be launched, with focus groups as the intial step at further investigating inpatient smoking rates, as well as attempting to reduce them in a more systemic way.

